# Duplex real-time reverse transcriptase PCR to determine cytokine mRNA expression in a hamster model of New World cutaneous leishmaniasis

**DOI:** 10.1186/1471-2172-11-31

**Published:** 2010-06-22

**Authors:** Claudia M Espitia, Weiguo Zhao, Omar Saldarriaga, Yaneth Osorio, Lisa M Harrison, Michael Cappello, Bruno L Travi, Peter C Melby

**Affiliations:** 1 Research Service, Department of Veterans Affairs Medical Center, South Texas Veterans Health Care System, 7400 Merton Minter, San Antonio, Texas, USA; 2 Department of Medicine, The University of Texas Health Science Center, 7703 Floyd Curl Drive, San Antonio, Texas, USA; 3 Department of Pediatrics, Yale School of Medicine, P.O. Box 208064. New Haven, Connecticut, USA; 4 Department of Microbiology and Immunology, The University of Texas Health Science Center, 7703 Floyd Curl Drive, San Antonio, Texas, USA

## Abstract

**Background:**

The Syrian hamster, *Mesocricetus auratus*, has distinct immunological features and is uniquely susceptible to intracellular pathogens. Studies in hamsters are limited by the relative unavailability of tools to conduct immunological studies. To address this limitation we developed duplex real-time reverse transcriptase (RT) PCR assays for the relative quantification of the mRNAs of hamster cytokines, chemokines, and related immune response molecules.

**Results:**

Real-time RT-PCR primers and probes were synthesized for analysis of interleukin (IL)-4, IFN-γ, TNF-α, IL-10, IL-12p40, TGF-β, IL-13, IL-21, chemokine ligand (CCL) 22, CCL17, Chemokine (C-C motif) receptor 4 and FoxP3 expression. Standard curves and validation experiments were performed for each real-time RT-PCR assay, allowing us to use the comparative Ct (2^-ΔΔCt^) method to calculate changes in gene expression. Application of the real-time RT PCR assays to a biological model was demonstrated by comparing mRNA expression in skin and lymph node tissues between uninfected and *Leishmania panamensis *infected hamsters.

**Conclusions:**

The duplex real-time RT PCR assays provide a powerful approach for the quantification of cytokine transcription in hamsters, and their application to a model of cutaneous leishmaniasis suggests that a balanced type 1 and type 2 cytokine response contributes to the chronic, nonprogressive course of disease. These new molecular tools will further facilitate investigation into the mechanisms of disease in the hamster, not only for models of leishmaniasis, but also for other viral, bacterial, fungal, and parasitic infections.

## Background

The Syrian golden hamster (*Mesocricetus auratus*) is highly susceptible to a number of human pathogens. The hamster has been described as a relevant experimental model that more representative of human disease than either the mouse or rat models for a number of human pathogens, including *Leishmania (Viannia) *spp. [1-4], *L. donovani *[5,6], *Trypanosoma cruzi *[7], *Entamoeba histolytica *[8], *Leptospira *and *Treponema *[9,10], hantavirus [11], Eastern equine encephalitis virus [12], Yellow Fever virus [13,14], West Nile virus [15], Nipah virus [16], hookworm [17], and various fungi and mycobacteria. Unfortunately, the use of the hamster in research has been hindered by the lack of commercially available immunological and molecular reagents to investigate mechanisms of disease.

The cloning and sequence analysis of portions of Syrian hamster interleukin 2 (IL-2), IL-4, gamma interferon (IFN-γ), tumor necrosis factor alpha (TNF-α), IL-10, IL- 12p40, and transforming growth factor beta (TGF-β) cDNA were reported previously [6], and these sequences have been used by a number of investigators to measure cytokine expression by northern blotting and conventional semi-quantitative RT-PCR [5,6,18,19]. More recently real-time quantitative reverse transcription-PCR assays for the quantification of cytokine mRNA expression using two-step SYBR green I RT-PCR protocols were used [20-22].

Studies in animal models of parasitic infections have shown that T lymphocytes and cytokines play a crucial role in the outcome of infection, both in terms of protective immunity and immunopathology. Of particular interest is the evidence that parasitic infections can trigger polarized CD4^+ ^T cell subset responses, and that cytokines produced by different Th1 or Th2 cells in the host can have opposing effects, resulting in either control of infection or promotion of disease [23]. There is enormous variety in the expression patterns of cytokine genes, i.e., they can be either constitutively expressed or can be activated or repressed when a cell is exposed to a particular signal [24]. Most of these responses appear to be controlled at the level of transcription and mRNA stability [22,25] so quantification by real-time RT PCR is widely used to investigate the immune response to various stimuli.

To better understand the hamster immune response to important pathogens, we determined the nucleotide sequence of additional hamster cytokines and chemokines (IL-13, IL-21, CCL17, CCL22), one chemokine receptor (CCR4), and one transcriptional factor (Foxp3). These cDNAs were chosen for study because of their role in a type 2 or regulatory T cell response, both of which have potential importance in the immunopathogenesis of leishmaniasis. This technical report describes the standardization of One Step (Taqman) Real Time PCR for the detection of these hamster mRNAs, and their application was validated in a model of localized cutaneous leishmaniasis.

## Results and Discussion

The Syrian hamster, because of its distinct immunological features and susceptibility to intracellular pathogens provides a unique opportunity for study of models relevant to human disease. However, studies in hamsters are limited by the relative unavailability of tools to conduct immunological studies. Previously, we identified sequences for several hamster cytokine cDNAs and used these to assess the immunological responses to infection using Northern blotting and conventional RT-PCR. We have now extended these studies by isolating and cloning a number of additional hamster cDNAs and developing and standardizing duplex real-time RT PCR assays for measurement of gene expression of 12 different mRNAs, which included cytokines, chemokines, a chemokine receptor and a transcription factor. While the measurement of protein levels has the most relevance to understanding immunopathogenesis and immunity, most cytokines/immune response genes are transcriptionally regulated, enabling the use of quantitative real time PCR to detect and quantify their expression. We were particularly interested in measuring the expression of these mRNAs because of their potential role in the immunopathogenesis of cutaneous leishmaniasis.

The newly isolated and cloned cDNAs included small portions of hamster IL-13, IL-21 and FoxP3, and the complete open reading frame (ORF) of the hamster CCL17, CCL22 and CCR4. All the cDNA sequences (excluding primers) were analyzed using Basic Local Alignment Search Toll (BLAST) [26] from the National Center from Biotechnology Information (NCBI) using the Nucleotide collection (nr/nt) database and the discontiguous megablast algorithm (intended for cross-species comparisons).

The source and size of the cloned cDNA and the percent identity of the sequence to the ortholog of other species are summarized in Table 1. Of note, three different cDNA clones containing the 1084 bp hamster CCR4 ORF were isolated from cDNA that had been reverse transcribed at 55°C from mRNA from lymph node tissue of a hookworm infected hamster. Like the other cloned cDNAs, each clone was sequenced twice in each direction. Three DNA polymorphisms were detected in positions 165, 363 and 695. Position 165 and 695 are predicted to make changes in the amino acid sequence. The real-time RT PCR primers and probe for CCR4 were designed to avoid the polymorphic positions. The primers and probes used for all of the real time PCR assays are shown in Table 2.

**Table 1:** Summary of Hamster cDNA sequences

Hamster Gene	Source of cDNA	Cloned cDNA fragment size	Percent nucleotide identity of hamster cDNA compared to ortholog ^1^				
			*Rattus norvegicus*	*Sigmodon hispidus*	*Peromyscus maniculatus*	*Mus musculus*	*Homo sapiens*
IL-13	Lymph node from hookworm infected hamster	177 bp	81%	85%	83%	80%	76%*

IL-21	Spleen from hookworm infected hamster	262 bp	90%	N/A ^2^	95%	92%	84%

CCL17	Hamster cDNA library	344 bp	89%	N/A	N/A	86%	77%

CCL22	Hamster cDNA library Lymph node from	310 bp	88%	N/A	N/A	89%	82%

CCR4	hookworm infected hamster Lymph node from	1084 bp	89%	N/A	N/A	90%	83%

FOXP3	hookworm infected hamster	581 bp	93%	N/A	92%	90%	85%

^1 ^Identity percentage of cross-species identity determined by Basic Local Alignment Search Toll (BLAST) from the National Center for Biotechnology Information (NCBI) using the Nucleotide collection (nr/nt) database and the discontiguous megablast algorithm in all instances except for comparison of hamster to human IL-13, where the Blastn algorithm for somewhat similar sequences was used. The percent identities excluded the primer sequences.

^2 ^N/A: sequences not available in NCBI.

**Table 2:** Hamster primers and probes used for duplex real-time RT PCR

*Hamster target gene*	*Primers and probes sequences*		*Amplicon length (bp)*
γ-actin	γactin-For	5'-ACA GAG AGA AGA TGA CGC AGA TAA TG-3'	70 bp
	γactin-Rev	5'-GCC TGA ATG GCC ACG TAC A-3'	
	γactin-Probe	5'-VIC - TTG AAA CCT TCA ACA CCC CAG CC-(TAMRA)-3'	

IL-10	IL-10-F	5'-GGT TGC CAA ACC TTA TCA GAA ATG-3'	194 bp
	IL-10-R	5'-TTC ACC TGT TCC ACA GCC TTG-3'	
	IL-10-P	5'-(6FAM) TGC AGC GCT GTC ATC GAT TTC TCC C-(TAMRA)-3'	

IL-4	IL-4-F	5'-ACA GAA AAA GGG ACA CCA TGC A-3'	95 bp
	IL-4-R	5'-GAA GCC CTG CAG ATG AGG TCT-3'	
	IL-4-P	5'-(6FAM) AGA CGC CCT TTC AGC AAG GAA GAA CTC C-(TAMRA)-3'	

IFN-γ	IFN-γ-F	5'-TGT TGC TCT GCC TCA CTC AGG-3'	130 bp
	IFN-γ-R	5'-AAG ACG AGG TCC CCT CCA TTC-3'	
	IFN-γ-P	5'-(6FAM) TGG CTG CTA CTG CCA GGG CAC ACT C-(TAMRA)-3'	

IL-13	IL-13-F	5'-AAA TGG CGG GTT CTG TGC-3'	81 bp
	IL-13-R	5'-AAT ATC CTC TGG GTC TTG TAG ATG G-3'	
	IL-13-P	5'-(6FAM)-TGG ATT CCC TGA CCA ACA TCT CTA GTT GC (TAMRA)-3'	

IL-21	IL-21-F	5'-GGA CAG TGG CCC ATA AAA CAA G-3'	80 bp
	IL-21-R	5'-TTC AAC ACT GTC TAT AAG ATG ACG AAG TC-3'	
	IL-21-P	5'-(6FAM)-CAA GGG CCA GAT CGC CTC CTG ATT-(TAMRA)-3'	

TGF-β1	TGFβ1-F	5'-GGC TAC CAC GCC AAC TTC TG-3'	81 bp
	TGFβ1-R	5'-GAG GGC AAG GAC CTT ACT GTA CTG-3'	
	TGFβ1-P	5'-(6FAM)-CCC TGT CCC TAC ATT TGG AGC CTG GA-(TAMRA)-3'	

CCL17	CCL17-F	5'-GTG CTG CCT GGA GAT CTT CA-3'	89 bp
	CCL17-R	5'-TGG CAT CCC TGG GAC ACT-3'5'-TGG CAT CCC TGG GAC ACT-3'	
	CCL17-P	5'-(6FAM)-CCA TTC CCA TCA GGA AGC TGG TGA TG-(TAMRA)-3'	

CCL22	CCL22-F	5'-TGG TGC CAA CGT GGA AGA C-3'	82 bp
	CCL22-R	5'-GAA GAA CTC CTT CAC TAC GCG C-3'	
	CCL22-P	5'-(6FAM)-CTG CTG CCA GGA CTA CAT CCG TCA GC-(TAMRA)-3'	

CCR4	CCR4-F	5'-GCT TGG TCA CGT GGT CAG TG-3'	80 bp
	CCR4-R	5'-GTG GTT GCG CTC CGT GTA G-3'	
	CCR4-P	5'-(6FAM)-TCC CTC CCA GGC CTC TTG TTC AGC-(TAMRA)-3'	

FOXP3	FOXP3-F	5'-GGT CTT CGA GGA GCC AGA AGA-3'	72 pb
	FOXP3-R	5'- GCC TTG CCC TTC TCA TCC A-3'	
	FOXP3-P	5'-(6FAM)-TTT CTC AAG CAC TGC CAA GCA GAT CAC C-(TAMRA)-3'	

TNF-α	TNF-α-F	5'-TGA GCC ATC GTG CCA ATG-3'	79 bp
	TNF-α-R	5'-AGC CCG TCT GCT GGT ATC AC-3'	
	TNF-α-P	5'-(6FAM)-CGG CAT GTC TCT CAA AGA CAA CCA G-(TAMRA)-3'	

IL-12p40	IL-12p40-F	5'-AAT GCG AGG CAG CAA ATT ACT C-3'	88 bp
	IL-12p40-R	5'-CTG CTC TTG ACG TTG AAC TTC AAG-3'	
	IL-12p40-P	5'-(6FAM)-CCT GCT GGT GGC TGA CTG CAA TCA-(TAMRA)-3'	

**Figure 1:**
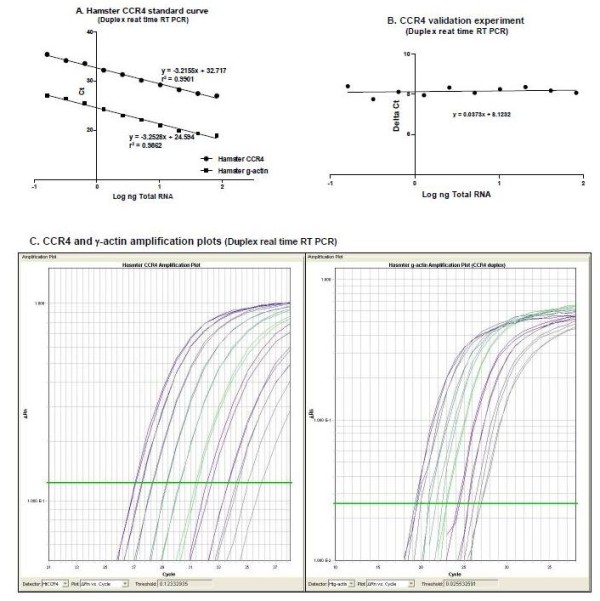
**Real-time RT PCR standardization and validation**. Standard curves (A), validation experiment (B) and amplification plots (C) for the hamster CCR4 duplex real-time Reverse Transcription (RT) PCR using TaqMan One-Step RT-PCR Master Mix, FAM-labeled probe for the target gene (hamster CCR4) and VIC-labeled probe for the endogenous control (hamster γ-actin).

For all the duplex RT-PCR assays the PCR product size was small (less than 200 bp), to limit the impact of infrequent RNA damage [27]. Because the amplification of the target gene and the reference gene (γ-actin) were made in the same reaction tube, the concentrations of primers and probes of both genes were optimized to avoid preferential amplification of the more abundant transcript [28] (see Table 3). For an optimal duplex reaction the amplification efficiencies of the target and reference genes should be approximately equal [29,30]. Therefore, validation assays were performed by amplification of the target and reference genes using serial dilutions of RNA. In each of the real-time RT PCR assays, the efficiencies were estimated for both the target and reference mRNAs by determining the slope (log input concentration versus the difference in Ct (target minus reference) [31]) of the lines using the formula E = 10^[-1/slope] ^[32,33]. Real-time amplification efficiencies of both target and reference genes of E = 2 are ideal [32]. To illustrate this, the amplification plots, standard curves and validation experiment data for CCR4 duplex assay, which are representative of all the genes studied, are shown in Figure 1. For the other assays the quality of the standard curves and amplification efficiencies can be judged from their slopes and correlation coefficients. In each case the absolute value of the slope of the line of each validation experiment was 0.1 or less (Table 4), indicating that the target and reference mRNAs were amplified with equal efficiencies.

**Table 3:** GeneBank accession number, primer and probe concentrations for hamster Duplex real-time RT PCR assays

Hamster target gene	GeneBank number	accession Specific forward and reverse primer concentrations (nM)	Specific probe concentration (nM)	γ-actin Primers concentration (nM)	γ-actin Probe concentration (nM)
IL-10	AF046210	700	100	80	80
IL-4	AF046213	650	100	65	100
IFN-γ	AF034482	600	100	100	100
IL-13	FJ548846	700	100	100	100
IL-21	FJ664142	600	100	100	100
TGF-β1	AF046214	600	100	150	100
CCL17	FJ664143	600	100	100	100
CCL22	FJ664144	650	100	100	100
CCR4	FJ664145,FJ664146,FJ664147	600	100	100	100
FoxP3	FJ664148	650	100	100	100
TNF-α	AF046215	600	100	100	100
IL-12p40	AF046211	750	100	100	100
γ -actin	FJ664149	-	-	-	-

**Table 4:** Real-time RT PCR Standard curves and validation experiments

*Hamster Duplex real-time RT PCR*	*Slopes of the standard curves*		*Correlation coefficient *(*r*^*2*^)		*Efficiencies*		Validation experiment (Absolute value)
		
	Target gene	γ-actin	Target gene	γ-actin	Target gene	γ-actin	
IL-10	-3.4094	-3.4745	0.95	0.99	1.96	1.94	0.065
IL-4	-3.0506	-31576	0.98	0.98	2.12	2.07	0.107
IFN-γ	-3.2861	-3.3459	0.93	0.93	2.01	1.99	0.059
IL-13	-3.6531	-3.6448	0.97	0.99	1.87	1.88	0.008
IL-21	-3.2268	-3.1831	0.98	0.98	2.04	2.06	0.043
TGF-β1	-3.4493	-3.3399	0.98	0.98	1.94	1.99	0.109
CCL17	-3.1135	-3.1023	0.99	0.98	2.09	2.01	0.081
CCL22	-3.2190	-3.2269	0.99	0.98	2.04	2.04	0.008
CCR4	-3.2155	-3.2528	0.99	0.98	2.04	2.02	0.037
FoxP3	-3.2598	-3.1920	0.98	0.99	2.02	2.05	0.077
TNF-α	-3.3286	-3.4026	0.99	0.99	1.99	1.96	0.074
IL-12p40	-3.4180	-3.4917	0.94	0.99	1.96	1.93	0.073

Standard curve slops, correlation coefficient (r2), efficiencies and validation experiment results for hamster Duplex real-time RT PCR assays. The efficiency estimation was calculated by E = 10^[-1/slope]^.

The equal efficiencies of amplification of the target and reference mRNA allowed for the comparative Ct (2^-ΔΔCt^) method to be used to determine the relative level of gene expression [34,35]. This mathematical model calculates changes in gene expression as a relative fold difference between an experimental and a calibrator sample and includes a correction for non-ideal amplification efficiencies [34]. Hamster BHK cells, which generally express cytokines and chemokines at a low level, were used as the external calibrator to enable the calculation of basal levels of gene expression in the uninfected hamster. In this way, we avoided having to assign the levels of mRNA expression in uninfected hamsters to the arbitrary value of 1, which enabled the variation in the uninfected group to be utilized in the statistical analysis. Because we used a relative method to calculate the gene expression, the numeric value of the fold change calculation depends on the Ct values of the normalizer and the calibrator used. With this approach, regardless of the calibrator used, the values of differences between the experimental groups are maintained. The low expression of the target genes in BHK cells allowed for the calculation of levels of expression such that the relative level of expression in the experimental tissue was always a positive value. Since the goal of these studies was to make comparison between the uninfected and infected tissue, determination of the absolute level of expression was unnecessary. The CT value for the target genes in BHK cells ranged from 32 to 40. For those genes that were expressed at a very low level (Ct = 40) in BHK cells, such as Foxp3, there is a high level of calculated expression in both the uninfected and infected tissue. Conversely, for the genes expressed more highly in BHK cells, such as TGF-β with a Ct = 32, the calculated level of expression in the hamster tissues is low. However, as noted above, this method is designed for the comparison of the two samples, in this case infected an uninfected tissue, and not for the absolute quantification of mRNA levels.

**Figure 2:**
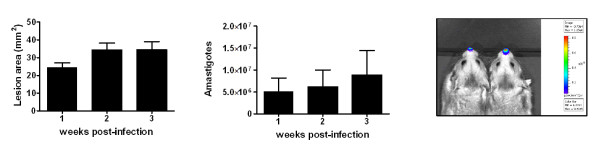
**Course of disease and parasite burden in *L. panamensis *infected hamsters**. 6-8 week old female hamsters (n = 6) with 3 × 10^6 ^luciferase (luc)-transfected *L. panamensis *promastigotes in the dermis of the snout. Lesion size was measured weekly and is shown as the lesion area (mm^2^) in panel A. The intralesional parasite burden was determined by in vivo imaging and is shown as the total intralesional amastigotes in panel B. Images of the luc-*L. panamensis *burden in two representative hamsters are shown in panel C.

We used a well-established model of localized cutaneous leishmaniasis caused by *L. panamensis *[1,3,4] to test the application of the standardized and validated duplex assays. In this model, at one-week post infection there is early evidence of a clinical lesion (inflammation and swelling) and parasites are easily identified in the tissue. Thereafter the lesion increases in size until 2-3 weeks post-infection when it is nodular with crusting and central necrosis (see Figure 2). After 3-4 weeks postinfection it transitions to a stable chronic lesion containing fewer parasites (data not shown). We investigated the immune response early in the course of infection because the innate and early adaptive immune responses are known to shape the clinical outcome and immunity in other models of *Leishmania *infection [36,37].

**Figure 3:**
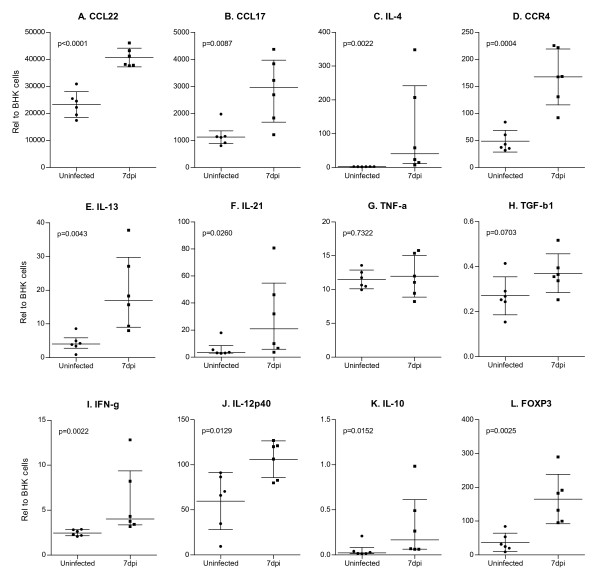
**Relative mRNA expression levels in skin from uninfected and 7-day *L. panamensis *infected hamsters**. Skin was harvested from the uninfected or infected snout at 7 days p.i. and the expression of multiple mRNAs determined by duplex real-time RT PCR. Results are expressed as a relative fold difference between experimental samples and BHK cells to which the value of 1 was arbitrarily assigned. For panels A, D, G, H, J and L the mean + SD are represented in the bar graph and statistical analysis utilized the unpaired t test. Data presented in panels B, C, E, F, I and K were not normally distributed so are shown as the median + interquartile range and the statistical analysis utilized the nonparametric Mann Whitney test. Each uninfected hamster is represented by a filled circle, and each infected hamster is represented by a filled square.

**Figure 4:**
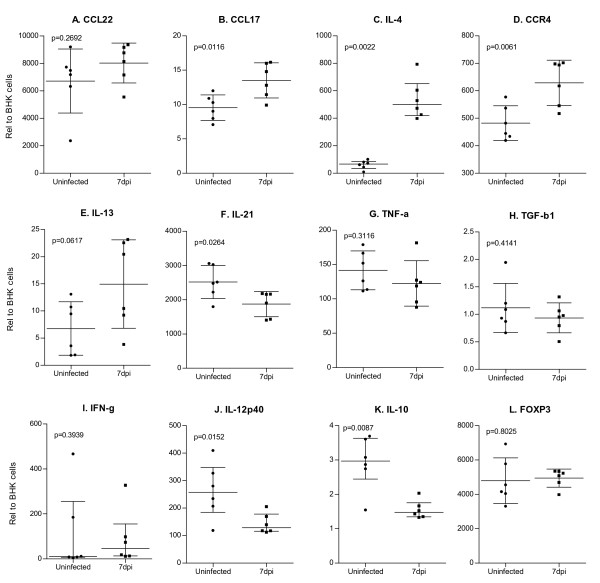
**Relative mRNA expression levels in lymph node from uninfected and 7-day *L. panamensis *infected hamsters**. Experiments were conducted as described for Figure 2. Results are expressed as a relative fold difference between experimental samples and BHK cells to which the value of 1 was arbitrarily assigned. For panels A, B, D, E, F, G, H and L the mean + SD are represented and statistical analysis utilized the unpaired t test. Data presented in panels C, I, J and K were not normally distributed so are shown as the median + interquartile range and the statistical analysis utilized the nonparametric Mann Whitney test. Each uninfected hamster is represented by a filled circle, and each infected hamster is represented by a filled square.

The role of type 1 (IFN-γ and IL-12p40) and type 2 (IL-4, IL-10, IL-13, and IL-21) cytokines in the immunopathogenesis of leishmaniasis is well known [38]. Our studies to dissect the immunopathogenic mechanisms in this model of LCL are ongoing, but several aspects of this preliminary work deserve comment. After relative quantification and normalization, we found that there were tissue-specific differences in the basal expression of several genes (see Figures 3 and 4). There was relatively greater basal expression in the LN compared to the skin for most transcripts (p ≤ 0.05 for IL-4, CCR4, IL-21, TNF-α, TGF-β, IFN-γ, IL-12p40, IL-10, and Foxp3). Conversely, the assays identified that basal expression of CCL22 and CCL17 mRNAs was significantly greater in the normal skin compared to the LN (*p*≤0.05), consistent with their prominent expression in Langerhans cells (LCs) and immature dendritic cells (DCs) [39]. At an early stage (1 week post-infection) there was concomitant upregulation of the type 1 (IFN-γ and IL-12p40) and type 2 (IL-4, IL-10, IL-13, and IL-21) cytokines at the site of cutaneous infection (Figure 2, *p *< 0.05 for all). This mixed cytokine response is reminiscent of what is seen in chronic nonhealing, but nonprogressive, *L. mexicana *infection in some strains of mice [40]. We also found that the levels of CCL22 and CCL17 mRNAs, which are strongly up-regulated upon DC maturation [39,41], were up-regulated at the cutaneous site of infection of hamsters infected with *L. panamensis *(Figure 3A and 3B; *p *< 0.05), consistent with the notion DCs are activated by *Leishmania *[42]. To our knowledge, this is the first demonstration of the induction of CCL17 and CCL22 expression in response to *Leishmania *infection. These chemokines have been implicated in the recruitment of activated Th2 cells and regulatory T cells, by signaling through the CC chemokine receptor 4 (CCR4) [43,44]. Consistent with this finding, at the site of cutaneous *L. panamensis *infection, we also found increased expression of Foxp3 (Figure 3L; p < 0.05), a transcription factor that is critical for the development and function of mouse CD4^+^CD25^+ ^regulatory T cells (Tregs) [45].

The sensitive real time RT PCR assays identified fewer infection-induced changes in gene expression in the LN compared to the skin at this early stage of infection. The response in the LN of infected hamsters was polarized toward a type 2 response with up-regulation of the type 2 cytokines (IL-4 (p < 0.05, Figure 4B-D) and IL-13 (in 3 out of 6 animals; non-significant)), down-regulation of IL-12 (Figure 4J; p < 0.05), and upregulation of the type 2 chemokine CCL17 and its receptor CCR4 (p < 0.05, Figure 4B-D). Further studies will be needed to determine if the more restricted cytokine expression in the LN compared to the skin is simply because there is shorter period of exposure to parasites in the LN, or because there are organ specific mechanisms that lead to distinct cytokine responses.

## Conclusions

The results presented here provide evidence that duplex real-time RT PCR is a powerful approach for the quantification of transcription immune response genes in the hamster model. Application of the assays developed here to a model of cutaneous leishmaniasis suggests that a balanced type 1 and type 2 cytokine response contributes to the chronic, nonprogressive nature of this disease. These new molecular tools will further facilitate investigation into the mechanisms of disease in the hamster, not only for models of leishmaniasis, but also for other viral, bacterial, fungal, and parasitic infections.

## Methods

### Experimental animals

Outbred Syrian golden hamsters (*Mesocricetus auratus*) were infected with hookworm as described previously [17]. Spleens and mesenteric lymph nodes (LNs) were harvested for isolation of mRNA and cloning of selected cDNAs. The model of localized cutaneous leishmaniasis was established by infecting 6-8 week old female hamsters (n = 6) with 3 × 10^6 ^luciferase-transfected [46]*L. panamensis *promastigotes (MHOM/CO/94/1989) in the dermis of the snout. Lesion size was determined by measuring the diameter in two perpendicular directions and calculating the area of the lesion in mm^2^. The intralesional parasite burden was determined by in vivo imaging as follows: Hamsters were anesthetized with anesthetic cocktail (ketamine, acepromazine, and xylazine) and 20 μL of luciferin (Gold Biotechnology, St. Louis, MO) at 1.5 mg/mL concentration were injected intradermally in the snout using a 31 G needle. Ten minutes later the animals were placed in prone position inside the chamber of an IVIS50 imaging system (Caliper, Life Sciences, Hopkinton, MA) and the luminometric images captured using medium binning and one minute exposure. The total parasite burden in the lesions was calculated by transforming photons/sec to parasite numbers using a standard curve of lesion-derived *L. panamensis *amastigotes obtained concurrently with the hamster evaluations. Animals used in this study were handled according to local and federal regulations, and research protocols were approved by our Institutional Animal Care and Use Committee.

### Cloning of hamster cDNAs

Hamster IL-13, IL-21, CCR4 and Foxp3 were cloned from 1 μg of total RNA isolated from lymph node or spleen tissue of a hookworm infected hamster [47]. The total RNA was reverse transcribed at 50 or 55°C using SuperScript^® ^III First-Strand Synthesis SuperMix (Invitrogen) and amplification was performed initially at a low annealing temperature (45°C), with extension at 72°C for 1.5 min and denaturation at 94°C for 1 min. Reactions that produced amplification products were repeated at incrementally (2 or 3°C) higher annealing temperatures (up to 58°C) until a single amplicon was achieved or all amplification products were lost. The hamster CCL17 and CCL22 cDNAs were cloned from 5 ng of plasmid DNA from a hamster cDNA library. Amplification was performed at 58°C annealing temperature, with extension at 72°C for 1.5 min and denaturation at 94°C for 1 min. The oligonucleotide primers used in the RT-PCR amplification for cloning of cDNAs were designed from regions of homology found among the corresponding published human, mouse, rat, and deer mouse cDNA sequences. In most instances, the use of multiple primer combinations was required to successfully amplify a specific product of the appropriate size. Degenerate primers were used when there was incomplete homology among the published sequences and when nondegenerate primers failed to yield an amplification product. The sequences of the primers used to successfully amplify those genes are as follows (5' to 3'):

IL-13: forward, gcagcatggtatggagcgtg; reverse, ccacttcrattttggtatc;

IL-21: forward, tastcatcttcttggggac; reverse ctttacatcttstggagctg;

CCL17: forward, aatcttcacctgcgttcctg; reverse, ctgcagtctcccaatgctc;

CCL22: forward, ggatgtaagtgcagcatggc; reverse, catcaggccttcttcaccag;

CCR4: forward, gatgaaccccacggatgtag; reverse, ttacaaagcgtcacggaggt;

Foxp3: forward, caaatggagtctgcaagtgg; reverse, gttgtggcggatggcatt.

The amplified cDNAs were cloned if they had a size similar to that predicted from the published homologous sequences and if it was the predominant amplification product of the reaction. In the case of hamster IL-13, two amplification products were cloned; the predominant amplification product was IL-13 genomic sequence including a intron-exon junction while the non-predominant amplification product corresponded to a small fragment of IL-13 mRNA. The amplification products were cloned into the pCR^®^2.1-topo plasmid using the TOPO TA cloning^® ^kit from Invitrogen according to the manufacturer's instructions. Each cDNA insert was sequenced twice in each direction with vector-specific primers at The Nucleic Acids Core Facility of The University of Texas Health Science Center at San Antonio.

### Real-time Primers and probes for mRNA quantification

Primers (forward and reverse) and TaqMan Fluorescent-labeled probes for real-time RT PCR assays were designed using Primer Express Software (Applied Biosystems, Foster City, CA) to specifically amplify hamster IL-4, IFN-γ, TNF-α, IL-10, IL- 12p40, TGF-β, IL-13, IL-21, CCL22, CCL17, CCR4, FOXP3 and γ-actin (GeneBank accession numbers in Table 3. The validated sequences of the primers and probes are listed in Table 2.

### RNA isolation and real-time RT Polymerase Chain Reaction

mRNA expression in 7-day infected and uninfected age/sex-matched control hamster tissues was determined by Duplex real-time RT PCR. After euthanasia of hamsters the snout skin and cervical lymph nodes were collected in RNA preservation buffer and frozen at -80°C (RNAlater; Ambion) until the RNA was isolated. Total RNA was extracted from 20 mg of tissue using the RNeasy kit (QIAGEN). All RNA samples were DNase treated with TURBO DNA-*free*™ kit (Ambion^®^) and quantified using a Thermo Scientific NanoDrop™ Spectrophotometer and kept at -80°C until ready to use. For Duplex real-time Reverse Transcription (RT) PCR of each target gene, 1 μl of RNA (10 ng/μl per sample or corresponding dilution for standard curves) were combined with a specific set of oligonucleotide primers, FAM-target gene-TAMRA labeled probe, γ-actin primers, VIC-γ-actin-TAMRA labeled probe, and 40X MultiScribe and RNase inhibitor mix in 25 μl of a total reaction volume using TaqMan One-Step RT-PCR Master Mix (Applied Biosystems). The PCR samples were subjected to an initial incubation for 30 minutes at 48°C (RT reaction) followed by 10 minutes at 95°C (AmpliTaq Gold pre-activation) and then, 40 cycles of 95°C for 15 sec and 60°C for 1 minute in an 7900 HT Fast Real-Time PCR System (Applied Biosystems).

Hamster γ-actin was used as an endogenous control to normalize differences in the amount of input RNA in each Duplex assay. Prior to acceptance of data for relative quantification, at least five standard curve dilutions for each Duplex assay were required. To validate equivalent efficiency of each target gene and γ-actin in the same duplex real time RT reaction, standard curves were generated from comparator RNA samples (expressing the gene of interest) made from decreasing (2-fold dilution) amounts of RNA. Once the Real Time RT PCR assays were validated, 10 ng of RNA from each experimental animal were used to quantify mRNA expression. The comparative Ct (2-ΔΔCt) method was used to study the experimental samples and RNA isolated from the BHK (hamster fibroblast) cell line was used as a calibrator.

### Statistical analysis

Differences in mRNA expression between non-infected animals and the animals 7- days post infection were analyzed by two tail Man-Whitney test or two tail unpaired ttest using GraphPad Prism version 5.01 for Windows, GraphPad Software, San Diego California USA, http://www.graphpad.com

## Authors' contributions

CME conducted cloning, real-time RT PCR design, standardization, validation and application experiments; WZ cloned cDNAs; OS designed real-time RT PCR primers and probes and provided expert discussion related to real-time RT PCR; MC and LMH conducted the hookworm infections and harvest of tissue. BLT conducted the hamster *Leishmania *infections. PCM provided direction and oversight to the entire project. All authors contributed to the experimental design and manuscript preparation, and all authors have read and approved the manuscript.
